# A long-time dataset of climate related events on Cyprus Island (1174–1913)

**DOI:** 10.1016/j.dib.2025.112007

**Published:** 2025-08-28

**Authors:** Emmanuel Eliot, Gilles Grivaud, Victor Beauvalet, Raphaëlle Krummeich, Armelle Couillet, Iris Charalambidou, Romain Reulier, Salih Gücel, Sebastien Rey-Coreyhourcq, Kyriakos Georgiou, Carole Nehme

**Affiliations:** a UMR CNRS 6266 IDEES, University of Rouen, rue Lavoisier 76821 Mont-Sain-Aignan Cedex, France; b UR 3831 – Groupe de Recherche Histoire, Université de Rouen – UFR des Lettres et Sciences Humaines, Rue Lavoisier, 76821 Mont-Saint-Aignan Cedex, France; c Near East University, Near East Boulevard, 99138, Nicosia / TRNC Mersin 10, Cyprus; d Department of Life Sciences, School of Life and Health Sciences, University of Nicosia, 2417, Nicosia, Cyprus; e UMR CNRS 6266 IDEES, Univerity of Caen, Esplanade de la paix, UFR SEGGAT, 14032 Caen, France; f University of Nicosia, 46 Makedonitissas Ave., P.O. Box 24005, 1700 Nicosia, Cyprus

**Keywords:** Cyprus island, Climate related-events, Societal vulnerability, Historical sources

## Abstract

The dataset aims to illustrate societal vulnerability to climatic conditions on the island of Cyprus between 1174 and 1913. It describes a dataset of events including epidemic and pest outbreaks, natural and social disasters. This dataset is unique for two reasons. Firstly, it is the most complete collection of events based mainly on secondary sources for the island, consisting of international and local historical sources dedicated to illustrating social vulnerability to climate. Secondly, it is a long time series (1174 to 1913) covering different periods of rule on the island. This study presents research material that contribute to better understand climate changes over time and build up a local long times series in a region where archives are scattered and less compiled in comparison with Western Europe.

Specifications Table

**Table:** 

<	Social sciences
Specific subject area	*Dataset of climate related events on the island of Cyprus between 1174 and 1913.*
Type of data	*Citations, references, raw data.*
Data collection	*Data were collected from primary, secondary and compiled sources. Numerized and written sources have been collected from different institutions, especially in Cyprus, Italy and France. Most of the sources are from compilation works (books, papers, archive catalogues and collections). Original sources (primary types) have also been collected by the authors. Systematic cataloging, transcription, if necessary, referencing and verification of the sources have been used to build the database. Table of events in CSV format with ID source and table of references according to the ID source are available.*
Data source location	*Country: Cyprus,* 35° 5′ 42.6912′' N and 33° 12′ 12.3480′' E UMR CNRS 6266 IDEES, University of Rouen, rue Lavoisier 76,821 Mont-Sain-Aignan Cedex, France
Data accessibility	Repository name: Nakala Data identification number: *(or DOI or persistent identifier)* Direct URL to data: https://nakala.fr/10.34847/nkl.3f7b46j5. Instructions for accessing these data: three files are provided 1) dataset description 2) database (IDref, year, place, event_type, IDsource, TypeSource) 3) data reference according to the IDSource (description, description in English, location of the source)
Related research article	*None*

## 1 Value of the Data

• This data paper can be considered as the first systematic exploration of a long-time dataset in Cyprus to figure out societal vulnerability to climate over time. It groups together 862 references of cited events which occurred on the island between 1174 and 1913. This collection covers mainly three historical periods of rule on the island (Lusignan, Venetian and Ottoman periods)
• The dataset includes primary, secondary and compilation sources that are generally scattered in different institutions, mainly in Southern Europe and the Middle East. The dataset compiles research works that were published by historians but also original data that were either not published or edited by regional publishers.
• The dataset includes 5 categories of events (weather events, plague/diseases events, natural events, locust events, starvation and food shortage events) which provide information about extreme disasters that affected the Cypriot societies for about 700 years. Most of these events are directly or indirectly connected to long-term climate variations.
• The dataset provides details on the sources, location, type of event and date of occurrence.
• The dataset contains information that provide a list of retrospective events that underly dynamic of climate variations and societal crisis for Cyprus island. Therefore, it may contribute to research areas such as archival studies, history, environmental and climate change studies

## 2 Background

Historical research exploring the links between past climates and human history dates back from the early 1960s [7,8,15,16]. Researchers explore information from archives that could be sensitive to climatic variations in human history such as harvest, grain prices, pest invasions, diseases, especially in Western and Central Europe. In Eastern Europe and the Middle East, archive sources focusing on such events are very scattered and less collected than in Western Europe. However, and for the Middle East region, some authors reconstruct the climate history from written sources based on climate disasters such as droughts, famine, infestation of locusts, mice, or rats [3,11] for the 11th to 14th centuries. The Grozfeld dataset on the Middle East [14], further integrated into the online platform tambora (tambora.org), includes data collected from Arabic sources, but covers very little information for non-Islamic regions. This is an important issue to consider for areas where Islamic society was not so dominant, or where colonisation by States or Empires lasted for a long time. In the Ottoman Empire and from a socio-political perspective, White’s contribution [15] on the impact of climate on societies is based on primary sources (miihimme defters). These records contain accounts of significant events from across the empire as gathered in the imperial centre. According to our knowledge [15] few local studies have been provided in the Middle East, especially by collecting exhaustively events in the same geographical unit over time. The island of Cyprus is particularly interesting as a small territory and as a place which was ruled by different empires (Lusignian Kingdoms (1192–1489), the Venetian empire (1489–1571), the Ottoman Empire (1571–1878) and the British Empire (1878–1960).

## 3 Data Description

**Fig. 1:**
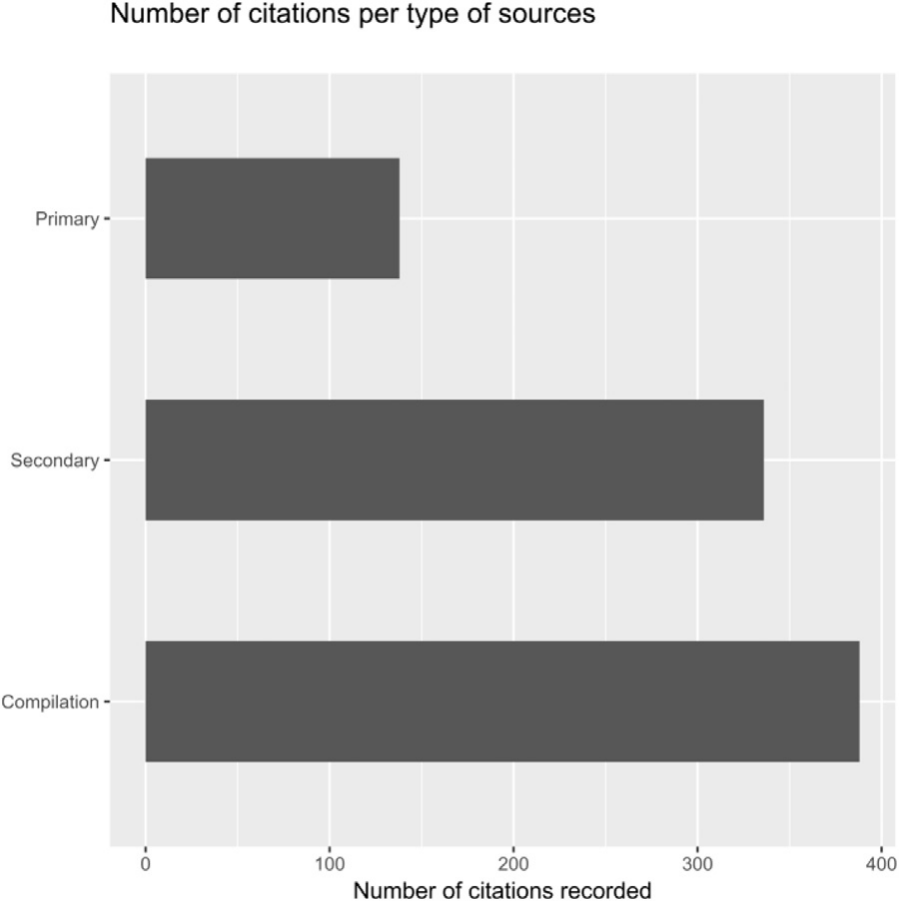
Types of sources collected.

The aim of the dataset is to collect and group data in order to deepen the data concerning climate-related events on Cyprus over time, in particular by including direct effects of climate variations (such as drought) or indirect consequences (such as starvation) or social consequences such as harvest. The data collection is mainly composed of published works or documents. These were divided into three main categories, i.e. primary, secondary sources and compilations, the last two categories represent the majority of the documents used in the collected data : almost 84 % of the 862 citations (Fig. 1).

• Categories of events in citations

**Fig. 2:**
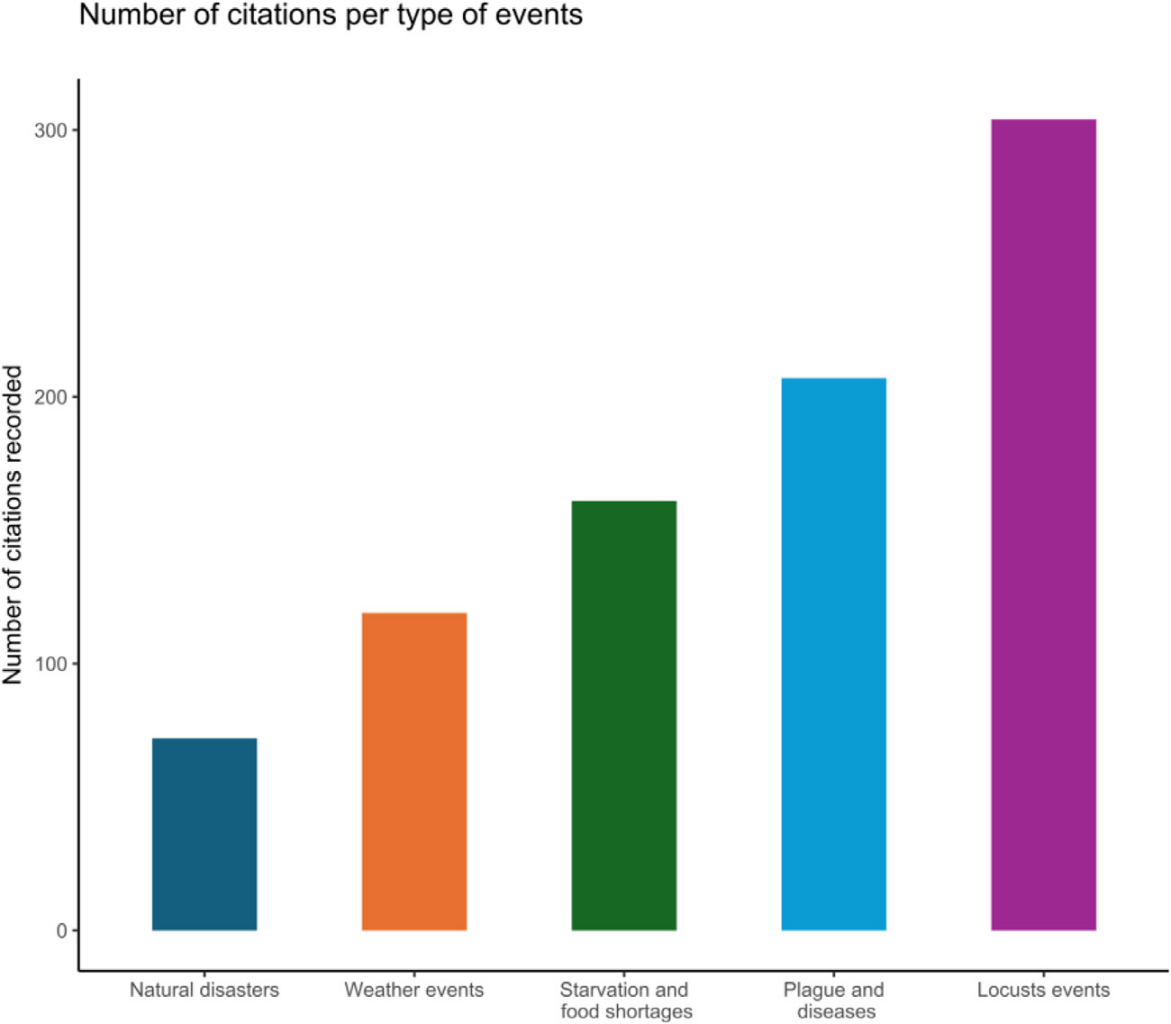
Categories of events in the database.

The data were coded in an Excel sheet and grouped into 5 categories (Fig. 2).

The ‘weather events’ category includes citations related to high-intensity hazards (heat, wind, rain, cold). The ‘natural disasters’ group includes hazards such as earthquakes and floods. The ‘locust events’ group includes citations about locusts and grasshoppers. Although these attacks were probably caused by different locust species which were predominant in the Mediterranean region [6], the sources do not mention differences because entomological differentiation was not developed in old sources. The same problem arises for the category of ‘plagues and diseases’. The citations of the plague probably refer both to the bubonic plague and to a nosological category that defines a great variety of transmissible diseases that are labelled 'plague’ because of their extreme virulence. From the 18th century and throughout the 19th century, some nosological categories appeared in the collected data (‘malaria', ‘cholera’), according to the evolution of the knowledge on types of symptoms. Therefore, the category ‘plagues and diseases’ brings together a great variety of transmissible diseases that were not properly defined or diagnosed until the end of the 19th century. Finally, a group called ‘starvation and food shortages’ is added to bring together events related to poor harvests and their consequences for food stocks.

• Historical coverage of the citations

**Fig. 3:**
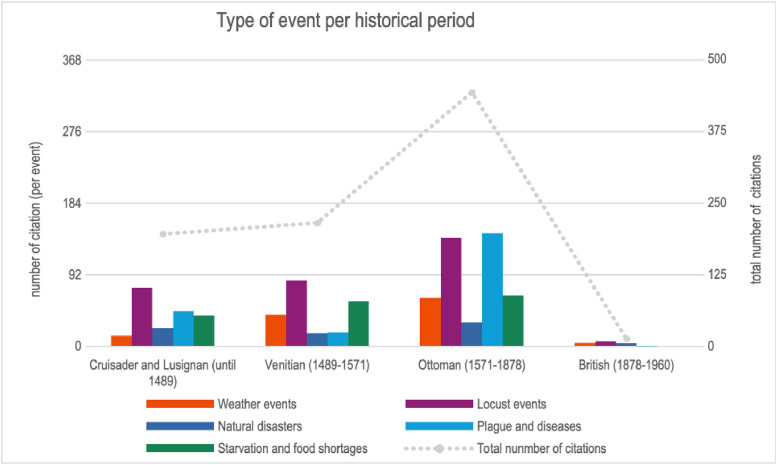
Distribution of event types per historical periods.

The dataset mainly covers three periods, for which citations are more frequent (Fig. 3). 51, 1 % of the citations concern the Ottoman period. The remainder concerns mainly two periods : the Crusader and Lusignan period (22,6 %) and the Venetian period (24,8 %).

The British colonial period is not fully covered, except for the very early period (35 years). Only 13 citations are collected, mainly on locust’s attacks. After the year 1913, the nature of the data and the sources are different. The British colonizers developed local statistical records (annual medical and sanitary report, for instance) and censuses, which have not yet been explored. These records are therefore primary sources, which unlike the other data are not based on the same method of collection and categorization.

In terms of category, locusts and plagues account for the majority of the citations (respectively 35,2 % and 24,1 % of the total). The second group concerns ‘weather events’ (13,8 %) and 'starvation and food shortages’ (18,6 %). The lowest proportion of citations is for ‘natural events’ (8,3 %). By period, the proportion of each category of events is quite close, except during the Ottoman period when the ‘plague and diseases events’ category reaches 32,9 % of the total number of events. On the contrary, the proportion of the latter is much lower during the Venetian period. As for the other periods, the proportion of 'weather events' is lower during he Crusader and Lusignan period.

• Frequency of citations and chronology of events

**Fig. 4:**
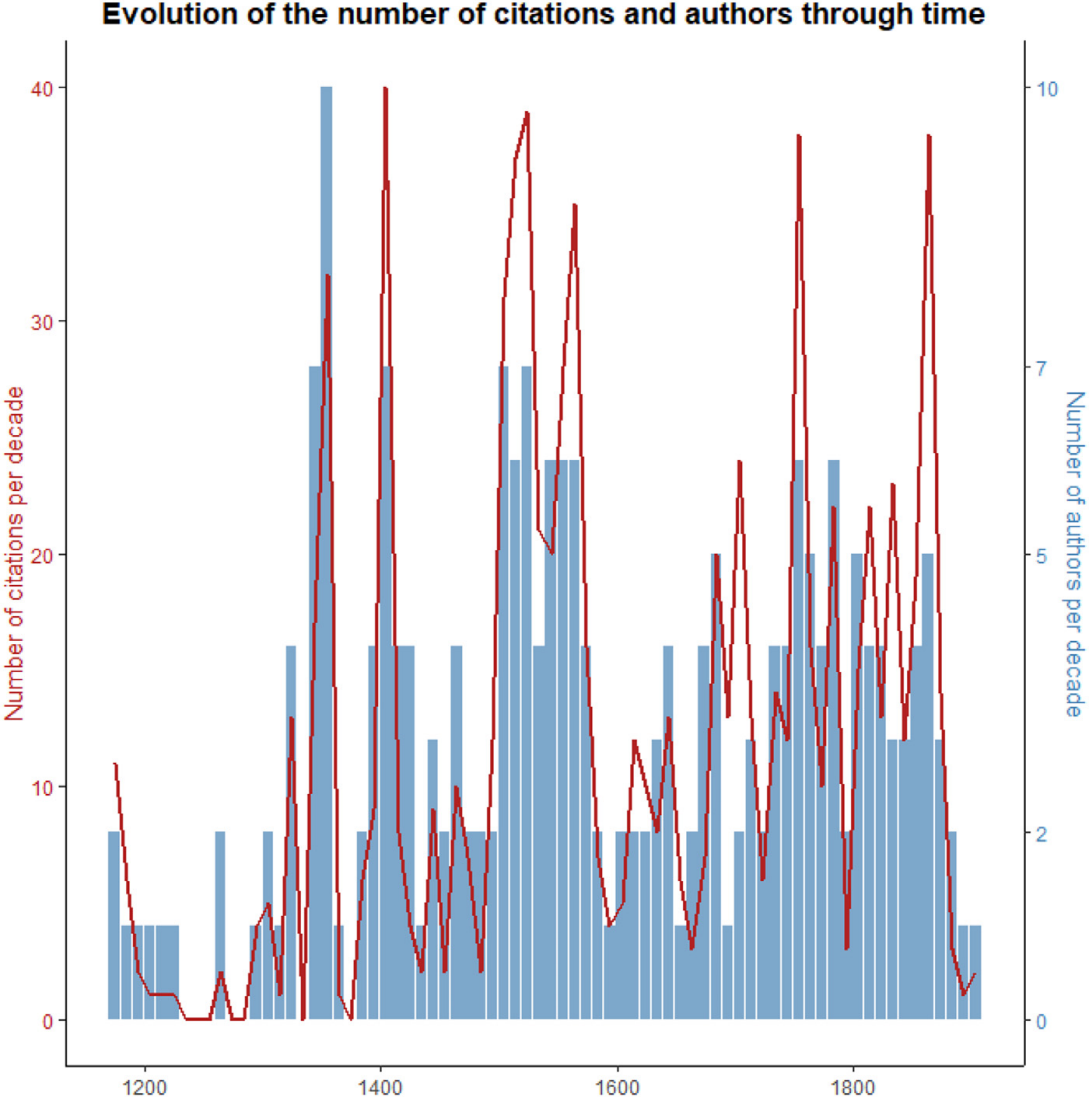
Comparison between the frequency of citations and the number of authors per decade.

When the citations are added by decades, 5 major peaks of citations are observed across the whole time interval under consideration (Fig. 4).

Two major peaks of citations are between 1500 and 1600. Peaks around 1400, 1750 and 1850 years are also observed. These peaks correspond to categories that are cited by different authors. More generally, a high frequency of citations corresponds to a high diversity of authors. For example in some decades between 1500 and 1600, more than 25 authors cited events that were sometimes reported more than 35 times. The high frequency of citation is interpreted as an indicator of better reporting, especially after the 16th century, or of an enhanced perception of the event. Therefore, the events dataset is built on the assumption that at least one citation counts as an event

**Fig. 5:**
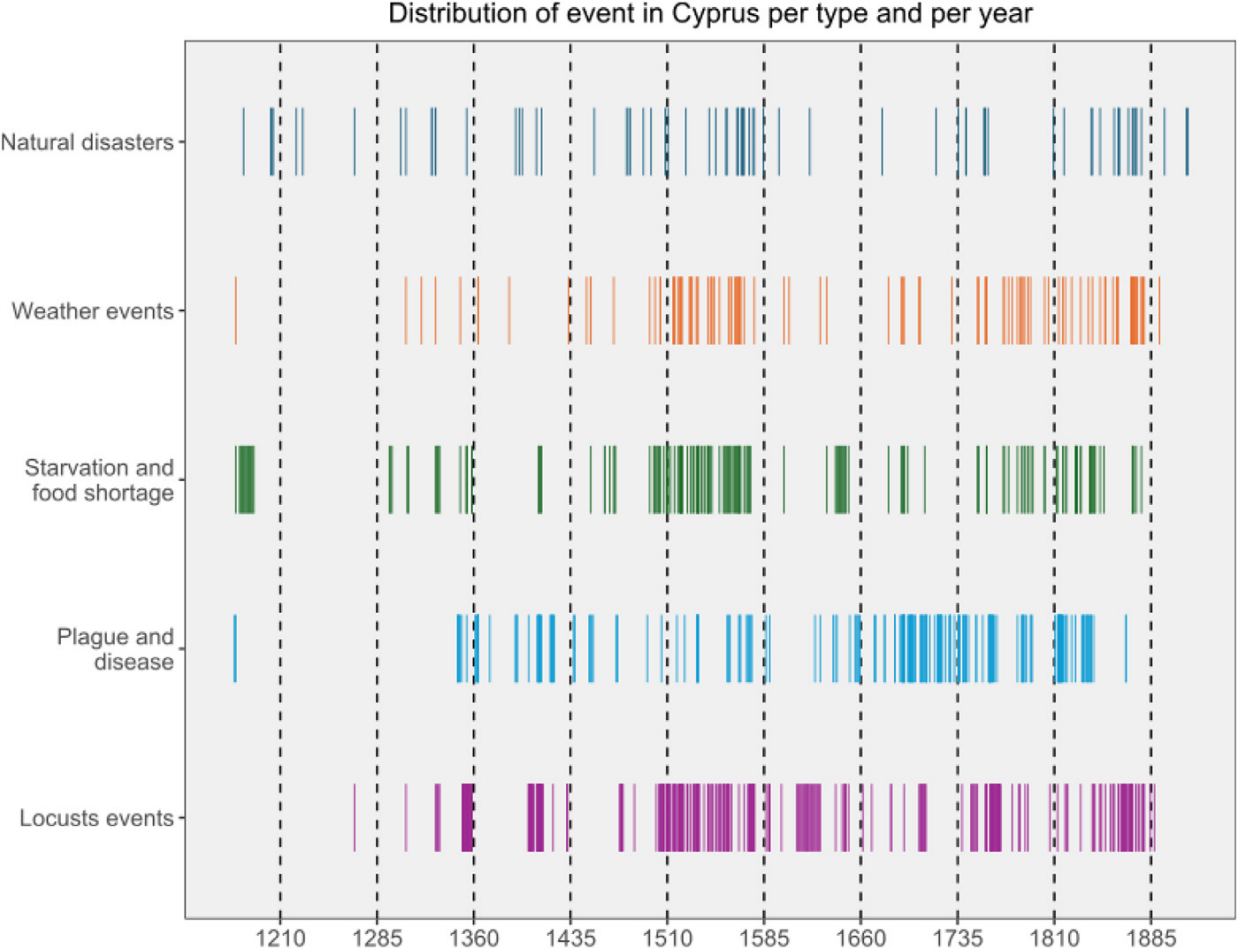
Visualisation of the types of events and their frequency between 1174 and 1913.

Apart from the differences in numbers between the categories, the distribution of events over time varies (Fig. 5). Clusters of events can be described for the different categories. ‘Starvation and food shortage’ events are particularly concentrated before the 13th century and more especially during the 16th century. Conversely, plague and diseases events are more frequently reported during the 17th and 18th centuries. ‘Locust’ events and ‘weather’ events have a quite similar pattern with clusters during the 16th and the 18th centuries. In addition, 36 % of the ‘locust’ and ‘starvation and food shortage’ events and one third of the plague and starvation and food shortage events occur within the same year. At least, the category of ‘natural disasters’ doesn’t seem to have a strong clustered distribution although reports are more frequent during the 16th century.

## 4 Experimental Design, Materials and Methods

**Fig 6:**
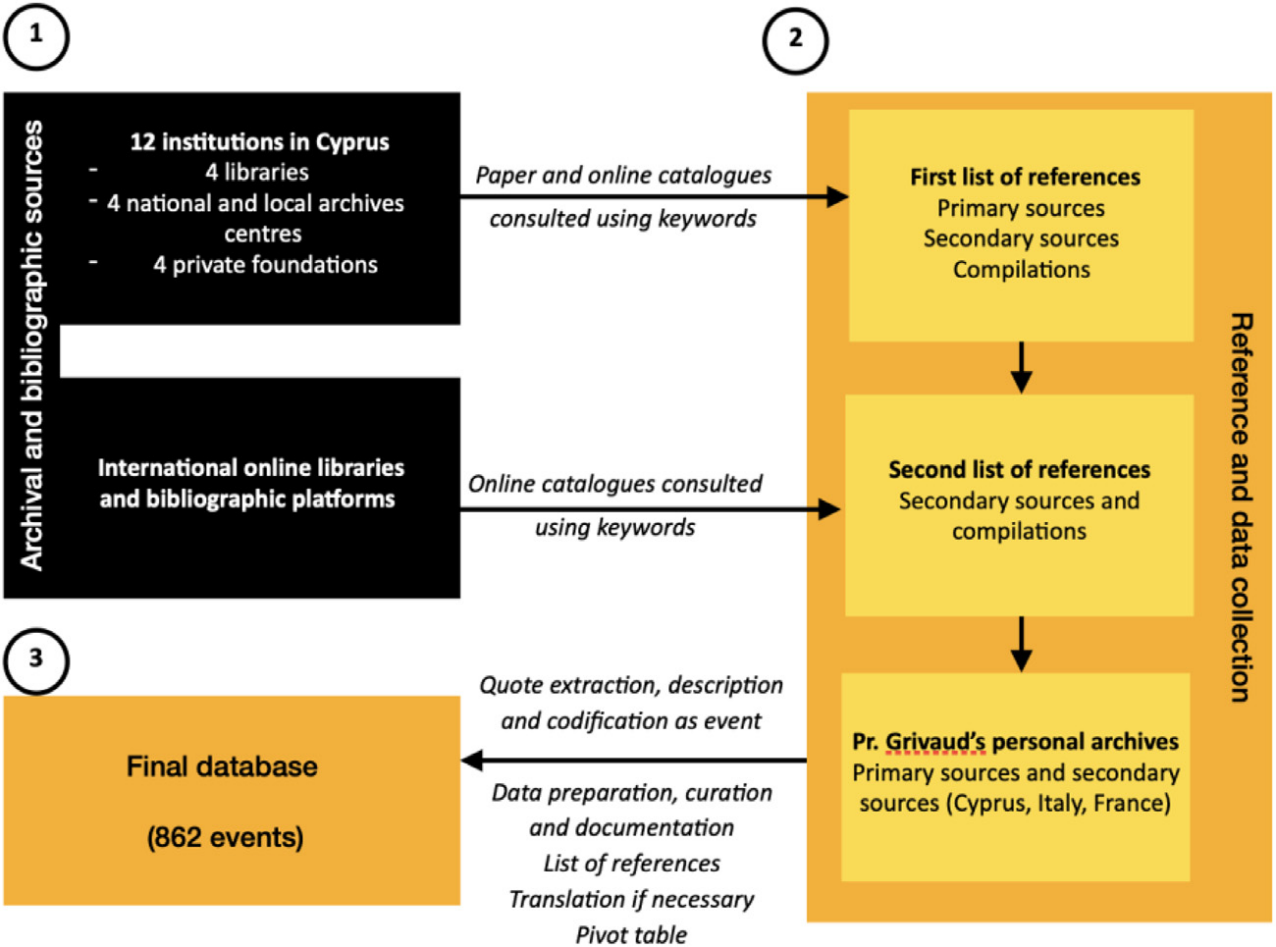
Flow-chart presenting the sources, the data collection and the methodology in order to produce the final database.

The objective of the dataset is to bring together available archive, paper and digital sources on Cyprus. This required an important effort to unearth pieces of information from different sources. The dataset brings together some primary, but mainly two different secondary sources. The first comes from historical works and research on Cyprus. They are published and are the main sources of information. The second comes from published works based on compilations, mainly collected outside of Cyprus (Fig. 6).

In order to build the dataset, environmental disasters that could be traced, such as diseases, pests floodings, droughts, and food shortages were investigated. These categories were chosen to examine the impact of climate and environmental changes. The keywords used offer a large spectrum of disasters that can be associated with climate variations. They are considered as robust indicators in order to investigate vulnerability of societies over time [1,2,9]. These words were used as a glossary in order to search the information in digital copies or via appendices in hardcopies. Translation in English or in French has been used for contemporary Greek materials. At least, Pr G. Grivaud [4], French specialist in the History of Cyprus, contributed to the dataset by using his own historical material, which was collected in European and Cypriot archives during his career. On the basis of this collection, the methodology used to construct the dataset consisted of coding each event cited and characterising it according to the year, the type of event, and the source. 85 % of the toponyms are ‘Cyprus’. The 15 % remaining are city or village names.

The dataset is mainly composed of European and Greek sources, although there are few original works based on Ottoman archives. Documents were available in English, French, Italian, Venitian , and Greek or translated either in English or in French by authors or researchers, or directly by us for the most recent sources. Much of the data was compiled or available from sources located in West European libraries or archives centers, as well as in Cyprus. In Cyprus, the 1973 partition reorganized the former British colony into two different political entities, where archives are divided into two systems with different policies. Documents and archives are scattered in different institutions, either ruled by the government or private founders. Data collection was carried out in the national and local structures, both governmental and religious. All the existing public and private archives of the island have been explored. One important question concerns the sources for the island during colonial occupation. Variations in the quantity and quality of data over time can affect the availability and the nature of the sources gathered. For instance, no systemic record of climate-associated events during the Ottoman colonization has been found in the Cypriot archives. Primary sources such as cadastral censuses and defters do exist in Cypriot archive centers, but these documents focus mainly on local tax impositions. Among those that have been translated [5], references to climate-related events are rare. However, the Ottoman State gave rights to the European consuls, especially for trade. Thus, information recorded by foreign consulates is a source for identifying events during the Ottoman period [10]. In addition, two original and external sources of description during the Ottoman period have been included in the dataset: the Ottoman sources of the national library in Sofia (Bulgaria) (1590–1880) [13] and the correspondences of the Orthodox patriarch in Jerusalem on Cyprus during the Ottoman period (1731–1884) [12]. These two sources were compiled in Greek language and have been translated into English.

## Limitations

Among the limitations to be mitigated, is the inclusion of data collection after 1913 (British colonial period) in the dataset. This will require more time and will be added in a next version.

Within the available dataset, data scientists, historians and climate change specialists might find important insights which would help to strengthen the dataset comprehensively 1) by completing the database with other types of data 2) by focusing on one of the periods 3) by using more disaggregated parts of the dataset in order to analyse the quality of event citations. A potential application of the dataset is undertaken in order to explore events occurrence within climatic variations (precipitation/temperatures) based on the measurement of natural archives (speleothems). The latter study is expected to assert the relevance of collecting such data for a better understanding of small-scale climatic variations. The dataset is a first step to fill the gap in data collection within the Eastern Mediterranean region, which is a ‘hotspot’ of the contemporary climate change observations. The aim is to better understand the dynamics and the history of past climate variations and their possible consequences.

## Ethics Statement

The authors have read and follow the ethical requirements for publication in Data in Brief and confirming that the current work does not involve human subjects, animal experiments, or any data collected from social media platforms.

## CRediT Author Statement

**Emmanuel Eliot**: conceptualization, investigation, resources, supervision, Writing - Original Draft **Gilles Grivaux**: investigation, resources **Victor Beauvalet**: data curation, resources, investigation, visualization **Raphaëlle Krummeich**: data curation, resources, investigation, visualization **Armelle Couillet**: investigation, resources **Iris Charalambidou**: investigation, resources **Romain Reulier**: investigation, resources **Salih Gücel**: resources, **Rey-Coreyhourcq Sebastien**: data curation **Kyriakos Georgiou**: resources **Carole Nehme**: funding acquisition, supervision, conceptualization.

## Data Availability

nakalaLong-term database of climate related events on Cyprus island (1174–1913) (original data).
